# Detection and characterization of rotavirus G and P types from children with acute gastroenteritis in Qom, central Iran 

**Published:** 2020

**Authors:** Saeed Shams, Seyed Dawood Mousavi Nasab, Hosein Heydari, Javad Tafaroji, Nayebali Ahmadi, Esmaeil Shams Afzali

**Affiliations:** 1 *Cellular and Molecular Research Center, Qom University of Medical Sciences. Qom, Iran*; 2 *Department of Research and Development, Pasteur Institute of Iran, Tehran, Iran*; 3 *Viral vaccine research center, Pasteur institute of Iran*; 4 *Pediatric Medicine Research Center, Qom University of Medical Sciences, Qom, Iran*; 5 *Proteomics Research Center, Department of Medical Lab Technology, Faculty of Paramedical Sciences, Shahid Beheshti University of Medical Sciences, Tehran, Iran*; 6 *Gastroenterology and Liver Diseases Research Center, Research Institute for Gastroenterology and Liver Diseases, Shahid Beheshti University of Medical Sciences, Tehran, Iran*

**Keywords:** Diarrhea, Rotavirus, Genotyping, acute gastroenteritis, vaccine, Iran

## Abstract

**Aim:**

The aim of the study is to estimate the burden of Rotavirus gastroenteritis as well as predominant genotypes of Rotavirus among children less than 5 years of age referring to Pediatric University Hospital in Qom, Iran.

**Background:**

Gastroenteritis is the fourth most common cause of death and accounts for 16% of all deaths in children <5 years of age worldwide.

**Methods:**

During two years, 130 patients referring to a pediatric hospital were enrolled in this study. After RNA extraction, Rotaviruses were detected by the VP6 gene. Then, G-typing (G1, G2, G3, G4, G8, G9, and G12) and P-typing (P4, P6, and P8) were performed using RT-PCR and specific primers.

**Results:**

The results of the PCR revealed that from a total of 130 patients, 22 cases (16.9%) showed positive VP6 by RT-PCR. G1 was mostly the predominant serotype (27%), accounting for 22% of all VP7-positive isolates, followed by G9 (18%), G2 (9%), G3 (9%), and G4 (9%). None of the strains revealed the presence of G8 genotype (0%), and 5 specimens (23%) were non-typable. The frequency of P typing was P8 (50%), P6 (23%), P4 (14%), and 3 samples were P-non-typable (13%), respectively. The dominant G-P combination was G1 [8] (32%).

**Conclusion:**

Such studies based on typing methods assists in the Rotavirus vaccine introduction by policymakers and design of new effective vaccines.

## Introduction

 Studies have generally shown that among infectious diseases, gastroenteritis is the fourth most common cause of death and accounts for 16% of all deaths in children <5 years of age worldwide (1, 2). It is estimated that only 20% of gastroenteritis is caused by bacterial agents, while the rest is caused by viruses. In epidemiological studies, diarrhea is usually defined as the passage of three or more loose or watery stools within 24 hours (3). The most significant viral agents as leading cause of acute gastroenteritis are Rotavirus, Calicivirus, Adenovirus, and Astrovirus. Rotavirus (RV) is known as the major agent of severe diarrhea among infants and young children with a high prevalence of morbidity and mortality worldwide (4, 5). The main clinical symptoms include watery diarrhea along with vomiting and fever which can result in dehydration, electrolyte imbalance, and death in young children below the age of 5 (6, 7). Rotaviruses belong to the family Reoviridae and based on RNA divergent sequence in VP6 region, they can be divided into eight groups named A–H (8). Species A rotavirus, which is a major cause of serious childhood dehydration gastroenteritis and has been reported to cause approximately a million deaths annually, mostly occurs in developing countries (3). These are non-enveloped agents with triple-layered capsid protein that surrounds a double-stranded RNA with eleven gene segments. The outer shell contains Rotaviruses composed of two proteins (elicit neutralizing antibody), the glycoprotein VP7 (G protein) and VP4 (P protein), which can further be differentiated into 23 G-type and 32 P-type, respectively (9, 10). Rotavirus strain entities may differ over time due to point mutations, genetic animal-human reassortment. 

Molecular techniques such as Reverse Transcription- PCR (RT-PCR) has enhanced our understanding of the diversity of Rotavirus strains (11). No specific treatment of RV infection is available and only supportive care for dehydration and its complications are recommended. It seems that vaccination is the most effective prevention strategy (12). It is estimated that expanded vaccination of the Rotavirus could prevent ~27 000 deaths in 2016 (13, 14). G1P8, G2P4, G3P8, G4P8, and G9P8 are the most common Rotavirus genotypes reported around the world (15). Thus, an increased understanding of the distribution of G and P genotypes strains circulating in different regions of the country is crucial before introducing rotavirus vaccines (16, 17). No study on the genotyping data has been reported in Qom city of Iran. So, the present work aimed to estimate the burden of Rotavirus gastroenteritis as well as predominant genotypes of Rotavirus among children less than five years of age referring to pediatric University Hospital in Qom, Iran. 

## Methods

**Patients and sample collection**

The study was approved by the Medical Ethics Committee of Qom University of Medical Sciences (IR.MUQ.REC.1394.129). After written informed consent was obtained from each participant or their parents, the patients joined the project. A total of 130 patients exhibiting symptoms of diarrhea admitted to Pediatric Hospital Medical Center, Qom, Iran (2017-2019) were enrolled in this study. They were suspected to have a viral infection and their stool exams were negative for RBC and WBC. Further, all tests for other enteric bacteria and parasites were negative. For Rotavirus characterization, the specimens were sent to the Cellular and Molecular Research Center of the university, and were stored at -80°C until use. The questionnaire was used for collecting all clinical, laboratory, and demographic characteristics.

**RNA extraction and cDNA Synthesis**

Fecal samples were prepared as a 10% (wt/vol) suspension of feces in 0.01 M phosphate-buffered saline (PBS; pH 7) and all samples were centrifuged at 5000×g for 5 min at 4°C, with the supernatants tested and then stored in sterile vials at −80°C for further study for RNA extraction. The supernatant was discarded and the viral dsRNA was extracted formed pellet by using CinnaPure Viral kit according to the manufacturer's protocol (SinaClon, Iran). cDNA synthesis was also accomplished using Biofact™ RT Series kit according to the manufacturer's instructions (Biofact, South Korea). The synthetized cDNAs were stored at -20°Cuntil analyzed by PCR. 

**Table 1:** Used primers and PCR conditions in this study

Primers	Sequences (53)		Target	PCR conditions			Amplicon size (bp)
	Forward	Reverse		Denaturation	Annealing	Extension	
Universal	GACGGVGCRACTACATGGT	GTCCAATTCATNCCTGGTG	VP6	94°C/60s	55°C/60s	72°C/60s	380
G typing	ATGTATGGTATTGAATATACCAC	AACTTGCCACCATTTTTTCC	VP7	94°C/60s	52°C/60s	72°C/60s	881
	CAAGTACTCAAATCAATGATGG		G1	94°C/60s	42°C/120s	72°C/60s	620
	CAATGATATTAACACATTTTCTGTG		G2				522
	ACGAACTCAACACGAGARG		G3				682
	CGTTTCTGGTGAGGAGTTG		G4				452
	GTCACACCATTTGTAAATTCG		G8				754
	CTTGATGTGACTAYAAATAC		G9				177
	CCGATGGACGTAACGTTGTA		G12				266
P typing	TATGCTCCAGTNAATTGG	ATTGCATTTCTTTCCATAATG	VP4	94°C/60s	50°C/60s	72°C/60s	663
		CTATTGTTAGAGGTTAGAGTC	P4	94°C/60s	45°C/120s	72°C/60s	362
		TGTTGATTAGTTGGATTCAA	P6				146
		TCTACTTGGATAACGTGC	P8				224

**Table 2:** Comparison of age, gender, and season between both Rotavirus-negative and Rotavirus-positive patients

Variable		All patients (n=130)	Rotavirus-positive patients (n=23)	Rotavirus-negative patients (n=107)	p-value
Age, Mean±SD (Month)		32.09±32.68	32.82±34.76	31.93±32.38	0.906
Gender	Male, N (%)	78 (60)	16 (69.6)	62 (57.9)	0.302
	Female, N (%)	52 (40)	7 (30.4)	45 (42.1)	
Season	Winter, N (%)	29 (22.3)	6 (26.1)	23 (21.5)	0.864
	Spring, N (%)	68 (52.3)	11 (47.8)	57 (53.3)	
	Summer, N (%)	33 (25.4)	6 (26.1)	27 (25.2)	

**Rotavirus RNA detection**

For detecting Rotavirus, the samples were evaluated using VP6-specific primers reported previously by WHO (Table 1)(18). PCR was performed in a 45 μl total reaction volume containing 4.5 μl of 10X PCR buffer, 2μl of 50mM MgCl_2_, 1 μl of 10mM dNTP, 5 µL of cDNA, 0.2 µL of TaqDNA polymerase 5U, 1 µl of primers for the VP6 gene (20pmol/μl), and 35.3μl of RNase-free H_2_O.

**Determination of G-P combinations**

The G-typing was done according to the WHO protocol for all positive cases from the previous step (18). The PCR was carried out using the consensus primers of the VP7 gene. Briefly, 5 µL of amplified fragments was used as a template for the second round by the primer combinations of VP7-R as well as G1, G2, G3, G4, G8, G9, and G12-F. The Nested-PCR reaction mixture was prepared in a 47µl volume containing 4.8 µl of 10X buffer, 2.5 µl of 50mM MgCl_2_, 1 µl of 10mM (each) dNTP, 0.2 µl of Taq polymerase 5U, 1 µl of each primer (20 pmol/µl), and 30.5 µl of nuclease-free H_2_O(18). The first-round of the P-typing was performed using a pair of specific primers of the gene coding for RotavirusVP4 protein. In a second-round PCR for P-typing and G typing is carried out under the same conditions (18).

**Statistical analysis**

The age of patients was expressed as the mean±SD. The student’s t and chi-square tests were used to determine significance of means for quantitative and qualitative variables, respectively. *P*-value < 0.05 was considered statistically significant. SPSS statistics software version 22 (IBM, NY, USA) was employed for all statistical analyses. 

## Results

Our study was performed within one year, where 130 stool samples were obtained from up to 15-year-old children with acute infectious gastroenteritis. The mean age of the patients was 32.09 months (standard deviation, ±32.68 months). Also, 60% and 40% of the patients were males and females, respectively. The results of the PCR showed that from a total of 130 patients, 22 cases (16.9%) were positive for VP6 by RT-PCR. Among them, males generally suffered more than females (15/7). The highest prevalence of Rotavirus was observed in the spring season (10/22, 45.5%). There were no significant correlations between age, gender, season, and the Rotavirus infection. 

The clinical symptoms in 22 Rotavirus-positive patients included vomiting (n=15 cases, 68.2%), fever (n=13 cases, 59.1%), and abdominal pain (n=8 cases, 36.4%). The results showed no significant difference in these symptoms between Rotavirus-positive and Rotavirus-negative patients (P > 0.05). There were no significant differences in WBC and RBC counts of stool and C-reactive protein (CRP) as well as erythrocyte sedimentation rate (ESR) of blood between the patients. G and P typing was done for 22 samples. Among different genotypes, G1 was mostly the predominant serotype (27%), accounting for 22% of all VP7-positive isolates, followed by G9 (18%), G2 (9%), G3 (9%), G4 (9%), G12 (5%). None of the strains revealed the presence of G8 genotypes (0%) and 5 samples (23%) were non-typable. 

**Figure 1:**
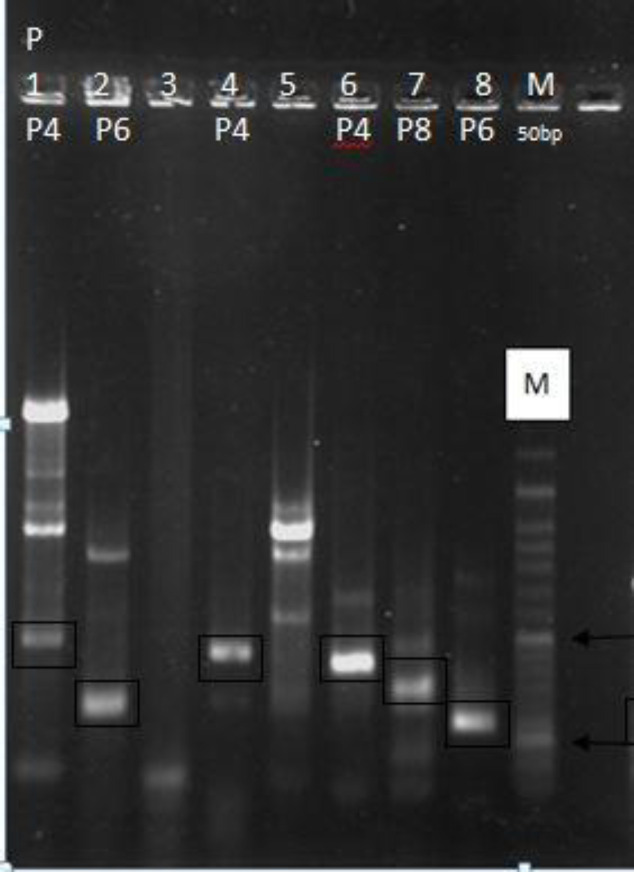
RT-PCR typing of group A* Rotavirus strains. *Lanes: M, DNA marker ladder; 1,4,6(P4); 2,8(P6); 7(P8) of VP4 genotype

As for VP4 genotyping (P type), the frequency of P type was P8 (50%), P6 (23%), P4 (14%), and 13% were P-non-typable respectively (Fig 1).

The dominant G-P combination was G1P [8] (32%), followed by G2P [4] (11%), G9P [6] (11%), and G3P [8] (11%). Further, G4P [6], G4P [8], G9P [4], G9P [8], and G12P [6] were detected in 25% of all evaluated specimens. In 10% of cases, no combination was observed.

## Discussion

Rotaviruses are the single most important cause of severe diarrhea in infants and young children worldwide with the average RVA positive value in Iran reported as 40.04% (15). The management of RVA circulating strains and the introduction of the RVA vaccine have a high priority for the healthcare system. Thus, it is critical to identify Iranian RVA strains and also to investigate the potential antigenic disparities between Iranian circulating and vaccine strains before introducing vaccines (19, 20). 

In the present study, using the RT-PCR method according to the WHO method, we were able to identify 22 cases of Rotavirus among 130 children (17%) under the age of 15 years with acute infectious gastroenteritis. The mean prevalence of Rotavirus was 78% in Shahr-e Kord and 79.2% in Tehran (21). Also, the prevalence of rotavirus reported by Moghim in Isfahan (2012) and Hassanzadeh in Shiraz (2001) was 12.6% and 11.3%, respectively (22, 23). Our findings are similar with a previous study conducted by Modares et al*.* who determined the prevalence of Rotavirus as 19% (24). Similar to results reported from different parts of Iran and other countries, diarrhea was the most common symptom observed in association with Rotavirus infection, followed by vomiting and fever. Our study also identified that Rotavirus infections were significantly higher in the spring season (10/22, 45.5%), which is in agreement with epidemiological studies in other regions of the world (25). According to gender data, the prevalence rate of disease was higher in boys than in girls (60%) which is in line with the findings of De Wit et al. study (26). 

Overall, G1, G2, G3, G4, and G9 were the five most common predominant genotypes worldwide. The current study revealed that the G1 type was mostly the predominant serotype (27%), followed by G9 (18%), G4 (9%), G3 (9%), G2 (9%), and G12 (5%) types which had a lower frequency. Numerous molecular epidemiological studies have shown that G1 is the most common circulating. Azaran et al., from southwest Iran showed that G1 and G2 are the most prevalent rotavirus genotypes (27). Farahtaj et al. determined that G1 was the most common type (28). Based on reports, the G2 has also been found to be common worldwide and the its prevalence has changed over time (29). These findings were different from the Khalili et al. reports on 200 subjects out of whom a prevalence 13% was found for G2 (30). In another study by Eesteghamati et al., the G2 genotype was reported in 5.5% of their cases (31). Unlike Kargar et al. report where G4 was dominant (32), our data showed that 9% of Rotavirus gastroenteritis were caused by this genotype. The prevalence of the G3 genotype was 24.6% reported by Motamedi‐Rad et al. (2017) (33), while our results showed a prevalence of 9 %among our patients. Furthermore, in this analysis, one G12 type was identified. In recent years, the spread of G12 seems to have accelerated further, particularly in combination with either P[8] or P[6] (34). Unlike the results found in Sierra Leone, Turkey, Morocco, and China (35-38), G8 genotype was not detected in the present study. In recent years, the increasing importance of the G9 type in many countries including Latin America, Brazil, and Iran has demonstrated(39). Due to the variable vaccine response and infectivity rate of the G9 type, this genotype has recently gained sufficient epidemiological concern worldwide. However, with the emergence of G9 and G12 Rotavirus genotypes, expectedly we have a number of gastroenteritis cases due to low heterotypic protection (15, 40). Different investigations in developing and industrialized countries have demonstrated a necessity for new generations of rotavirus vaccines to include G9 strains (39, 41). The P genotyping analysis also showed that the isolated Rotavirus strains with the P8 made up 50% of the cases, followed by P6 and P4 types with rates of 23% and 14%, respectively. Mousavi-Nasab et al. showed (2019) that P8 as the dominant genotype for VP4 gene (42). The results of P and G combination in this study revealed that G1 [8] genotype was dominant with the prevalence of 32%, followed by G2 [4] (11%), G9 [6] (11%),and G3 [8] (11%). G4 [6], G4 [8], G9 [4], G9 [8], and G12 [6].

Our result is in agreement with previous studies by Lorestani et al. who showed G1P8 as dominant genotype with a prevalence rate of 57.8% (43). In our study, 13.7% of detected RV was non-typable, and that was similar to a previous study by Kargar et al., who reported it as 12.5% in Jahrom, south Iran (32). 

In summary, this is the first report of determining rotavirus strains in Qom city of Iran. Nevertheless, large-scale study and continuous surveillance appear to be necessary. Such studies assist in rotavirus vaccine introduction by policy makers and designing new effective vaccines.
